# Investigating the role of mitochondrial DNA D-loop variants, haplotypes, and copy number in polycystic ovary syndrome: implications for clinical phenotypes in the Chinese population

**DOI:** 10.3389/fendo.2023.1206995

**Published:** 2023-09-06

**Authors:** Yang Chen, Wei-jia Wu, Li-wei Xing, Xiao-juan Zhang, Jing Wang, Xiao-yan Xia, Rui Zhao, Rong Zhao

**Affiliations:** ^1^ Nanjing University of Chinese Medicine, Nanjing, Jiangsu, China; ^2^ Department of TCM (Traditional Chinese Medicine), Hainan Women and Children’s Medical Center, Haikou, Hainan, China; ^3^ Department of Scientific Research, Hainan Women and Children’s Medical Center, Haikou, Hainan, China; ^4^ Yunnan University of Chinese Medicine, Kunming, Yunnan, China

**Keywords:** PCOS, mtDNA D-loop, haplotype, mtDNA copy number, polymorphic loci, mutation

## Abstract

**Background:**

The presence of genetic variations in mitochondrial DNA (mtDNA) has been associated with a diverse array of diseases. The objective of this study was to examine the correlations between mtDNA D-loop, its haplotypes, and polycystic ovary syndrome (PCOS) in the Chinese population, and the associations between mtDNA D-loop and symptoms of PCOS. The study also sought to determine whether the mtDNA copy number in Chinese patients with PCOS differed from that of individuals in the control group.

**Methods:**

Infertile individuals who only had tubal or male factor treatment were the focus of research by The Cancer Genome Atlas (TCGA) and Gene Expression Omnibus (GEO). mtDNA haplotypes were categorized using polymorphic D-loop sites. mtDNA D-loop, PCOS features, and mtDNA haplotypes were analyzed using R software to determine the strength of the association between the three. There are certain DNA haplotypes linked to PCOS. Microdroplet digital polymerase chain reaction (PCR) was used to determine the mtDNA copy number in a convenience sample of 168 PCOS patients and 83 controls.

**Results:**

Among the research group, the majority of D-loop mutations were infrequent (frequency< 1%), with only 45 variants displaying a minimum allele frequency (MAF) of 5% or higher. No association was found between polymorphism loci in PCOS patients and body mass index (BMI). Noteworthy, C194T, 1A200G, 523delAC, and C16234T showed positive correlations with elevated LH/FSH levels. Additionally, specific polymorphic loci G207A, 16036GGins, and 16049Gins within the D-loop region of mtDNA potentially exerted a protective role in PCOS development. Conversely, no statistical significance was observed in the expression levels of C16291T and T489C. Chinese women with mtDNA haplotype A15 exhibited a decreased risk of developing PCOS. Moreover, a significant difference in mtDNA copy number was detected, with controls averaging 25.87 (21.84, 34.81), while PCOS patients had a mean of 129.91 (99.38, 168.63).

**Conclusion:**

Certain mtDNA D-loop mutations and haplotypes appear to confer protection against PCOS in Chinese women. In addition, elevated mtDNA copy number may serve as an indicator during early stages of PCOS.

## 1 Background

The prevalence of polycystic ovarian syndrome (PCOS) among women of reproductive age is estimated to range between 6.3% and 8.5% (1). This condition is characterized by ovulatory dysfunction, insulin resistance (IR), and hyperinsulinemia, and a complex interplay of endocrine factors, including hyperandrogenism, irregular menstrual cycles, and the presence of polycystic ovaries as confirmed by ultrasonography (1).

The findings of hormonal studies indicated that women with PCOS exhibited distinct hormone levels in comparison to women in the control group. In particular, women with PCOS exhibited elevated levels of thyroid-stimulating hormone (TSH), total testosterone (T), and the ratio of luteinizing hormone (LH) to follicle-stimulating hormone (FSH), while experiencing decreased levels of FSH, thyroxin (T4), and progesterone (1, 2). Elevated concentrations of cholesterol, low-density lipoproteins (LDL), very-low-density lipoproteins (VLDLs), and triglycerides (TGs) are commonly observed in women with PCOS, while levels of high-density lipoproteins (HDLs) and HDL-cholesterol (HDL-C) tend to be diminished (3, 4).

The etiology of PCOS remains inadequately comprehended (5). Some reports imply that dysregulation of various RNA modifications and some long non-coding RNAs (lncRNAs) dysfunctions have been observed in women with PCOS (6) (7). This poses a threat, since certain PCOS samples have also shown altered M^6^ RNA modification and its regulators (5), which are involved in tumor growth (8). A similar dysregulation (GAS5 overexpression) may be seen in the expression of lncRNAs in PCOS samples (7); these lncRNAs interact with other mRNAs (9), miRNAs (10), and proteins in the cytoplasm (11).

In addition, the oxidative stress has been recently recognized as a contributing factor in the etiology of PCOS (12). The exclusion of a role for mitochondria in the pathogenesis of PCOS is not feasible, as they serve as a crucial site for the generation of reactive oxygen species (ROS) and play a central role in various metabolic processes, including the biosynthesis of steroid hormones (13, 14).

MtDNA and other constituents of the mitochondria are exceptionally susceptible to damage caused by ROS due to their abundant production within the mitochondria.

The rate of mutations in the mitochondrial genome is higher compared to that in nuclear DNA, rendering it more vulnerable to oxidative damage. The aforementioned phenomenon can be attributed to its proximity to the electron transport chain (ETC) apparatus, which produces ROS, the lack of histones that safeguard DNA, and the limited efficacy of DNA repair mechanisms. The mtDNA displacement loop (D-loop) is the only non-coding region present in the mitochondrial genome. According to Stoneking (15), the hyper-variable region-1 (HVR1) (np 16024-16383) and hyper-variable region-2 (HVR-2) (np 57-372) are recognized as significant sites for acquired mutations (15). The D-loop serves as the central regulatory site for the replication and transcription processes of mtDNA, as it contains the origin of replication for the leading strand and promoters for both the heavy and light strands (16). Mutations in this region may lead to an increase in the formation of cellular ROS and subsequent oxidative stress. This, in turn, can have an effect on the replication and transcription of mtDNA. A number of human disorders, such as PCOS, have been associated with sequence abnormalities in the mtDNA D-loop (17–20). Undoubtedly, the presence of mutations and polymorphisms in mtDNA can give rise to compromised ATP synthesis and heightened generation of ROS, culminating in oxidative stress and impairment of mitochondrial function. The involvement of oxidative stress in the development of polycystic ovary syndrome (PCOS) is well-established, as it contributes to the occurrence of insulin resistance, hyperandrogenism, and follicular dysfunction. There is a prevailing belief that these substances have the potential to interfere with the production of energy in mitochondria and elevate levels of oxidative stress, thereby exerting an impact on the development of follicles, steroid hormone synthesis, and insulin sensitivity (21, 22).

Additionally, the quantity of mtDNA copies plays a significant role in determining the level of mtDNA transcripts expressed within a cell (23). In typical physiological conditions, the cellular content of mtDNA remains relatively constant in order to meet the energy requirements necessary for cell survival (24). The alteration of mtDNA replication quantity is hypothesized to be associated with increased susceptibility to various diseases (25, 26). The aforementioned modifications are instigated by environmental oxidants and the interplay between genes and the environment. The association between changes in mtDNA copy number and the development of various diseases, such as PCOS, has been established in previous research (27). The investigation of D-loop changes and potential abnormalities in mtDNA copy number has been the subject of several studies on PCOS, yielding thoughtful findings (28). The analysis of mtDNA has been conducted to identify single nucleotide polymorphisms (SNPs), which enables the classification of distinct mitochondrial haplogroups. According to van Oven and Kayser (29), East Asians possess a significant proportion, approximately 50%, of the global mitochondrial haplogroups. These haplogroups specifically include A, B, D, G, M7, M8, M9, N9, and R9 (29). Various diseases, such as PCOS, have been associated with specific mtDNA haplogroups and variations. This association also extends to individuals of East Asian descent (30, 31). Consequently, the present study focused on the Han Chinese population residing in Eastern China in order to investigate the potential association between variants and haplogroups in the mtDNA D-loop region and PCOS. The primary objective was to identify susceptibility factors specific to this ethnic group that may contribute to the development of PCOS. In addition, an examination was conducted to compare the mtDNA copy number between individuals with PCOS and a control group. This analysis aimed to offer novel perspectives on the underlying mechanisms of PCOS.

## 2 Methods

### 2.1 Ethical considerations

The data used in this study were obtained from publicly available databases, and therefore, ethical approval and informed consent were not applicable. However, the original studies from which the data were derived are expected to have obtained ethical approval and informed consent in accordance with the guidelines and principles outlined in the Declaration of Helsinki.

### 2.2 Study population and sample collection

The study population comprised a cohort of Han Chinese individuals residing in Eastern China. The inclusion criteria focused on infertile patients diagnosed with PCOS, allowing for the investigation of potential associations between variants and haplogroups in the mtDNA (mtDNA) D-loop region and PCOS. At least two of the following phenotypic characteristics are necessary for a PCOS diagnosis: the presence of 12 or more small 2–9 mm follicles in each ovary or increased ovarian volume>10 mL on ultrasound examination (2); clinical or biochemical signs of hyperandrogenism (hirsutism, acne, or serum androgen>2.64 nmol/L); and (3) polycystic ovarian morphology. Patients having a history of disease or therapy that might alter basal hormone levels (such as congenital adrenal hyperplasia, Cushing’s syndrome, or androgen-secreting tumors) were also excluded.

Control individuals were selected from patients experiencing infertility due solely to tubal or male factors, without any concurrent endocrine disorders leading to anovulation or elevated androgens.

### 2.3 Data retrieval and clinical data collection

Gene Expression Omnibus (GEO) database (https://www.ncbi.nlm.nih.gov/geo/) and UCSC Xena database (https://xena.ucsc.edu) were mined for information on polymorphic loci and haplotypes in the mtDNA D-loop region (32). The homeostasis model assessment of insulin resistance (HOMA-IR) was calculated as part of the clinical data gathered. FINS (U/mL) FPG (MMOL/L)/22.5 was used to calculate HOMA-IR (33). Subgroups of people with PCOS have been diagnosed using BMI, LH/FSH ratio, testosterone (T), and HOMA-IR values (34, 35). The PCOS subgroups included both normal-weight and overweight/obese people, those with a low LH/FSH ratio and those with a high one, those with normal T levels and those with hyperandrogenemia, and those with either normal insulin metabolism or insulin resistance.

### 2.4 Screening and haplotyping of SNP loci in the mtDNA D-loop region

Base mutation sites and variations were identified by aligning sequences from the mtDNA D-loop region from the control region panel to the Cambridge reference sequences (36). MitoTool (37) was used for the primary haplotype analysis. In order to verify and further investigate the haplotypes that were generated, we used HaploGrep, an online mitochondrial haplotype analysis tool available through the human mitochondrial genome resource MITOMAP (https://www.mitomap.org/). Types having a quality score of 80% or above were included in the final haplotype analysis (38). If a sample’s haplotype quality score was below 80%, it was either subjected to further extraction and sequencing or excluded from the study.

### 2.5 mtDNA copy number analysis

Individuals with PCOS and a control group were compared in order to learn more about the connection between mtDNA copy number and PCOS. The 168 PCOS patients and the 83 healthy controls all had peripheral blood samples taken. Following the manufacturer’s protocol, total DNA was extracted using a commercial DNA extraction kit. Utilizing primers specific to the mitochondrial gene of interest (e.g., ND1) and a nuclear reference gene (e.g., β-actin), the mtDNA copy number was calculated. In order to determine the relative mtDNA copy number, the Ct values of the mitochondrial gene were compared to those of the nuclear reference gene.

### 2.6 Statistical analysis

Statistical analysis was conducted using R software. The strength of the association between mtDNA D-loop variants, haplotypes, and PCOS features was assessed using appropriate statistical tests, such as chi-square tests or logistic regression analysis. The correlation between specific polymorphic loci and clinical parameters (e.g., LH/FSH levels) was evaluated using correlation analysis. Differences in mtDNA copy number between the PCOS group and the control group were analyzed using t-tests or non-parametric tests, depending on the distribution of the data. p-values<0.05 were considered statistically significant.

## 3 Results

**Figure 1:**
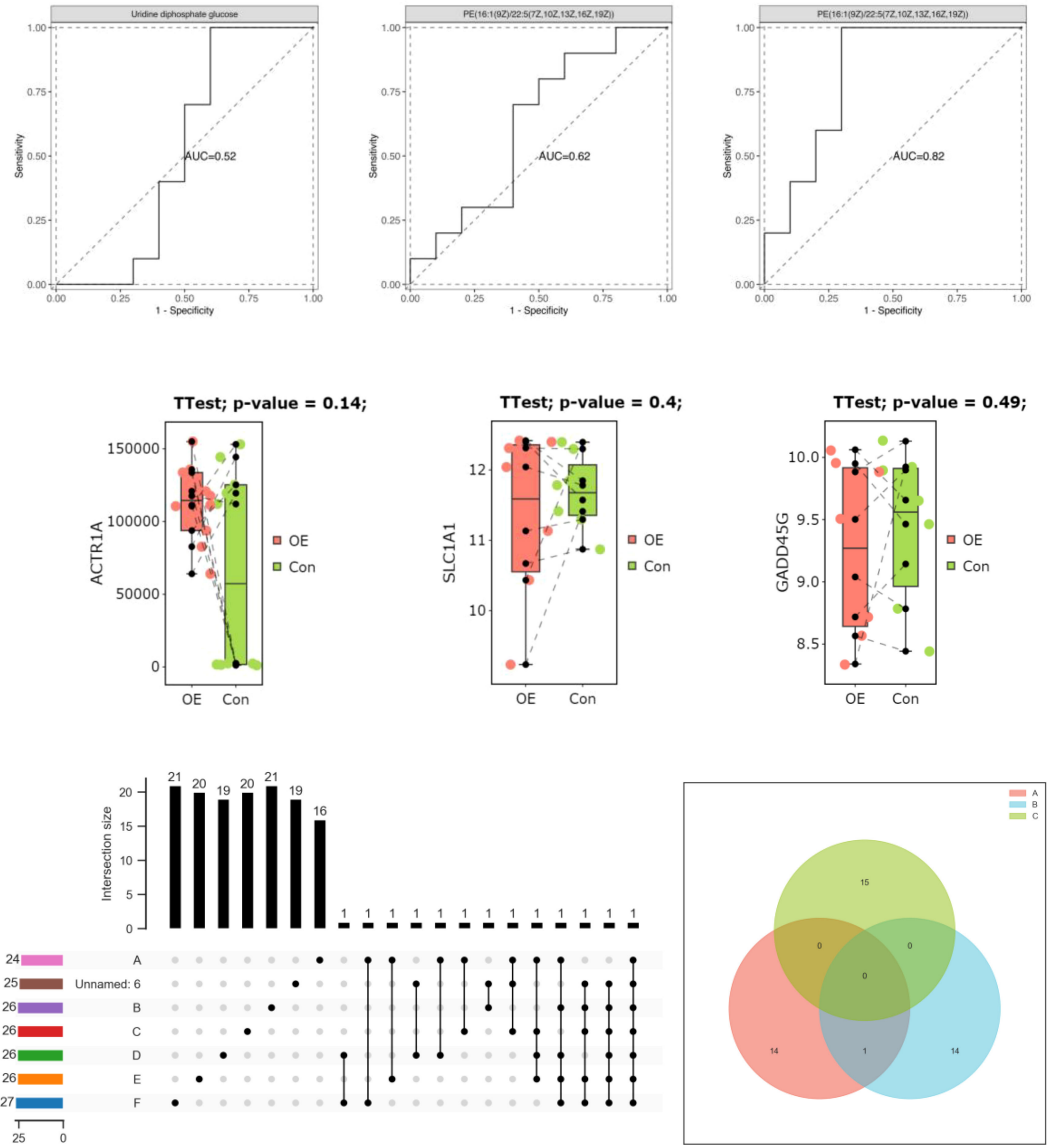
Comparison of clinical data between PCOS and control.

This study included a cohort of 422 patients diagnosed with PCOS (PCOS), with an average age of 27.53 ± 3.27 years. Additionally, a control group of 409 individuals without PCOS, with an average age of 27.90 ± 3.29 years, was also included in the study. There was no significant difference observed in age (p=0.067) and PRL levels (p=0.745) between individuals with PCOS (PCOS) and the control group. The results indicate that there was a statistically significant increase in fasting insulin levels (p<0.001), as shown in **Table 1** and **Figure 1**.

**Table 1:** Comparison of clinical information between PCOS and control.

Index	PCOS group	Control group	p
Number of cases	422	410	
Age	27.53 ± 3.27	27.90 ± 3.29	0.067
BMI	24.04 ± 3.71	22.23 ± 3.44	<0.002
FSH (m IU/mL)	6.34 ± 1.37	7.02 ± 1.52	<0.002
LH (m IU/mL)	11.92 ± 6.07	4.95 ± 2.19	<0.002
T(nmol/L)	1.89 ± 0.87	1.50 ± 0.79	<0.002
PRL (ng/mL)	16.01 ± 9.72	16.29 ± 9.66	0.745
FPG (mmol/L)	5.53 ± 1.45	5.29 ± 0.44	0.009
FINS (m U/L)	15.86 ± 9.21	10.28 ± 4.87	<0.002

**Figure 2:**
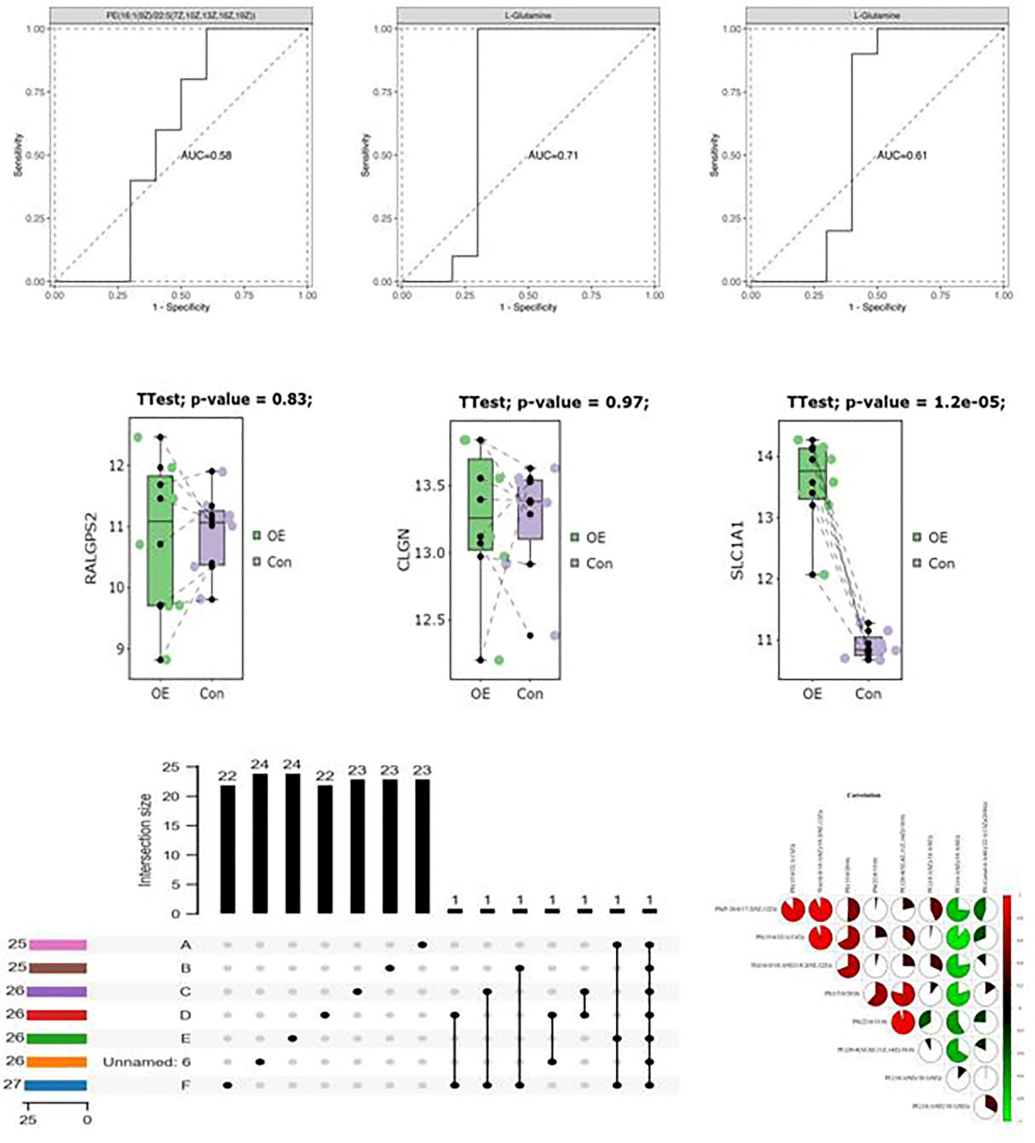
Correlation analysis between mtDNAD-loop region polymorphism and PCOS.

To assess the significance of mitochondrial D-loop region variants in relation to PCOS (PCOS), a comparative analysis was conducted between the reference Cambridge Reference Sequence (RCRS) and 505 nucleotide variants. These variants encompassed 61 base insertions, 28 base deletions, and 416 base substitutions. Due to the relatively low occurrence (<1%) of D-loop variants within the study population, only 45 variants were selected for correlation analysis in this study. These variants were chosen based on a minimum allele frequency > 5% (MAF > 5%), comprising of 35 base substitutions, 8 insertions, and 2 deletions. Please refer to **Table 2** and **Figure 2** for further details.

**Table 2:** Correlation analysis of polymorphic loci in D-loop region of mtDNAwith PCOS.

SNP_S_	PCOS group (n=421)	Control group (n=409)	Before correction	P	After correction a	P	$P_{BH^{b}}$
	n (%)	n (%)	OR (95%CI)		OR (95%CI)		
C150T	80 (18.77)	104 (25.19)	0.687 (0.493–0.956)	0.027	0.712 (0.505–1.001)	0.052	0.327
G207A	12 (2.62)	29 (6.86)	0.366 (0.179–0.743)	0.006	0.321 (0.152–0.674)	0.004	0.046
A263G	419 (99.29)	398 (98.56)	3.493 (0.954–12.78)	0.060	5.189 (1.297–20.754)	0.021	0.217
16036Gins	25 (5.71)	43 (10.27)	0.529 (0.314–0.890)	0.017	0.537 (0.313–0.920)	0.025	0.217
16036GGins	3 (0.49)	23 (5.39)	0.085 (0.020–0.359)	0.002	0.081 (0.018–0.349)	0.002	0.024
16049Gins	3 (0.49)	37 (8.81)	0.050 (0.012–0.207)	<0.002	0.052 (0.012–0.218)	<0.002	<0.002
C16234T	18 (4.05)	31 (7.34)	0.533 (0.288–0.980)	0.044	0.583 (0.312–1.084)	0.089	0.441
T16362C	170 (40.15)	197 (47.93)	0.728 (0.554–0.959)	0.025	0.735 (0.552–0.975)	0.034	0.249

**Figure 3:**
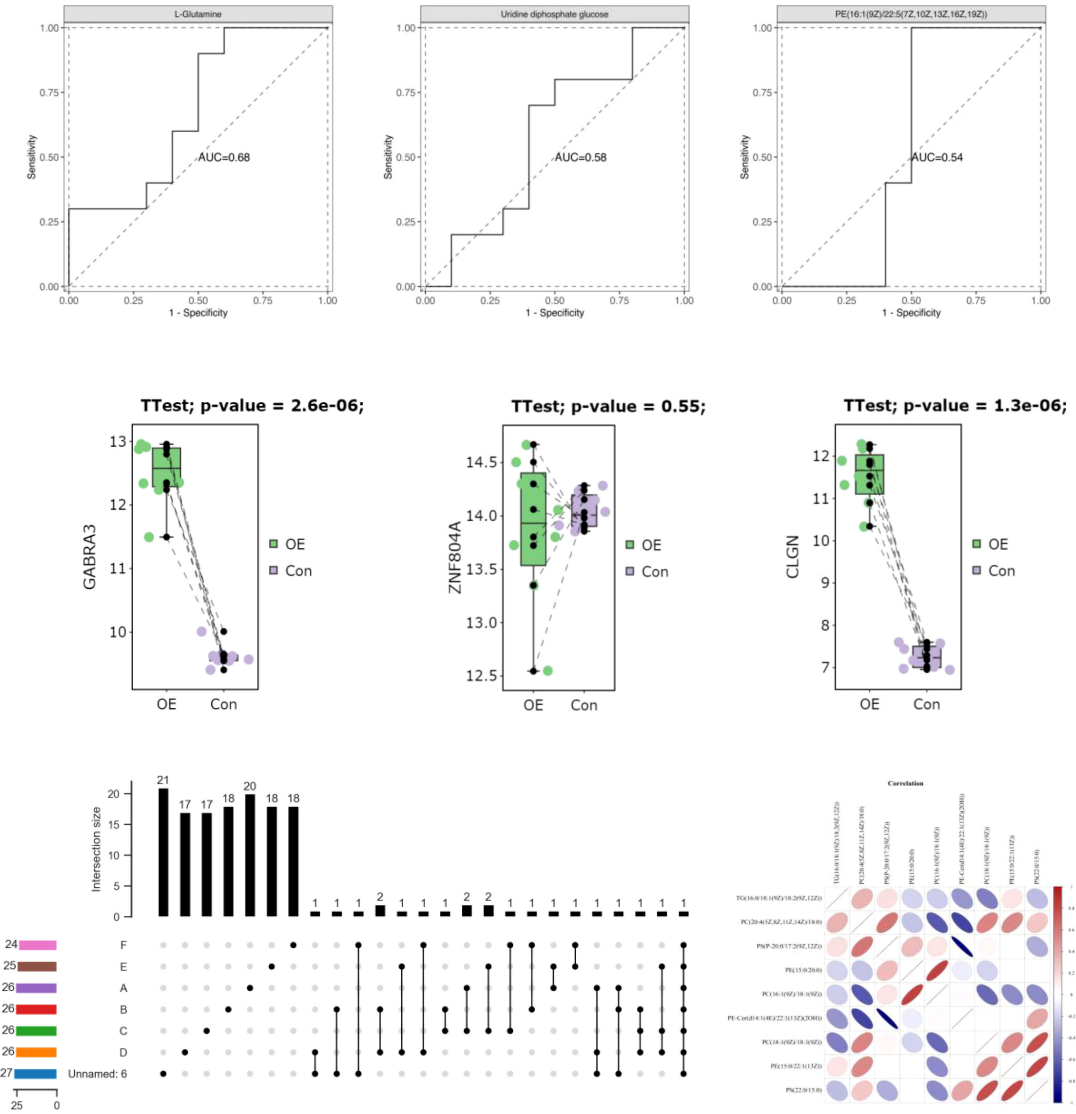
Association analysis of polymorphic loci in D-loop region with BMI levels.

The study involved the categorization of patients with PCOS (PCOS) into two groups based on their body weight, namely, normal weight body mass index (BMI) (BMI<24) and overweight/obese (BMI≥24), using the classification criteria of Chinese body mass index. The aim was to compare the variations in polymorphic loci within the D-loop region between these two groups. The analysis of **Table 3** and **Figure 3** revealed a lack of correlation between polymorphic loci and the level of BMI in patients diagnosed with PCOS (PCOS).

**Table 3:** Correlation analysis between polymorphic sites in D-loop region and BMI level.

SNP_S_	BMI<24 (n=213)	BMI ≥ 24 (n=208)	Before correction	*P*	After correction a	*P*	$P_{BH^{b}}$
	n(%)	n(%)	OR (95%CI)		OR (95%CI)		
T152C	68(31.46)	49(23.08)	0.655(0.425–1.008)	0.055	0.663(0.429–1.023)	0.064	0.643
A16183C	63(29.11)	45(21.15)	0.654(0.419–1.020)	0.062	0.651(0.416–1.016)	0.059	0.643
C16261T	17(7.51)	28(12.98)	1.838(0.958–3.520)	0.068	1.841(0.961–3.528)	0.067	0.643

**Figure 4:**
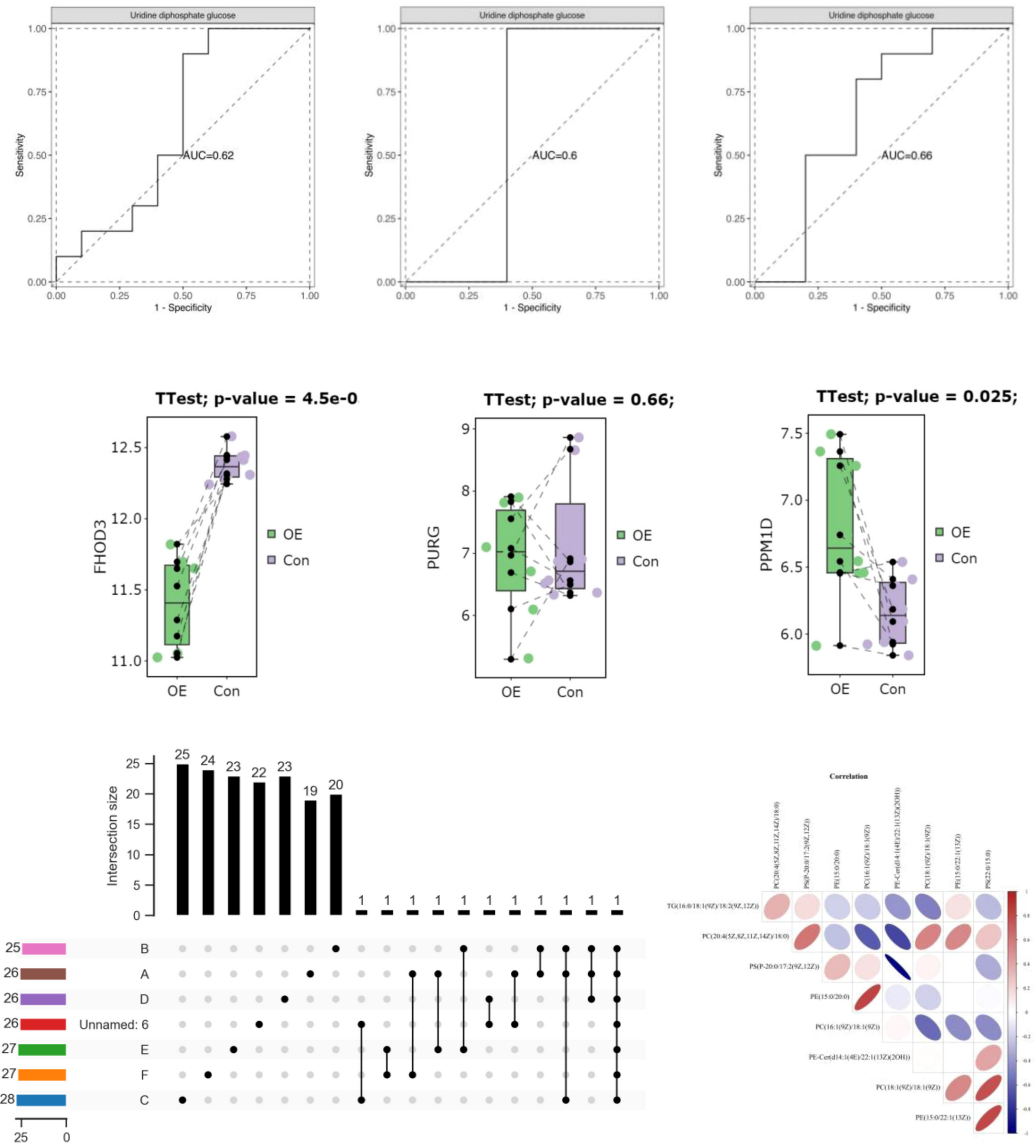
Association analysis of polymorphic loci in D-loop region with LH/FSH.

The patients diagnosed with PCOS (PCOS) were categorized into two groups based on their LH/FSH ratio: a group with a normal LH/FSH ratio (LH/FSH 2) and a group with a high LH/FSH ratio (LH/FSH>2). Subsequently, the variations in polymorphic loci within the D-loop region were compared between these two groups. The genetic variants C194T, 1A200G, 523delAC, and C16234T were found to be correlated with elevated levels of LH/FSH. After adjusting for age and body mass index (BMI), the observed associations continued to be statistically significant. Nevertheless, following the application of false discovery rate (FDR) correction, the observed association between these polymorphic loci and high LH/FSH ratios was no longer statistically significant (refer to **Table 4** and **Figure 4**).

**Table 4:** Association analysis of polymorphic loci in D-loop region with LH/FSH.

SNP_S_	LH/FSH ≤ 2 (n=258)	LH/FSH >24 (n=163)	Before correction	*P*	After correction a	*P*	$p_{BH^{b}}$
	n(%)	n(%)	OR (95%CI)		OR (95%CI)		
C194T	5(1.55)	10(5.52)	3.712(1.124–12.256)	0.032	3.595(1.070–12.076)	0.040	0.284
A200G	4(1.16)	10(5.52)	4.969(1.325–18.63)	0.018	4.467(1.177–16.949)	0.029	0.284
523delAC	98(38.37)	82(49.69)	1.587(1.067–2.359)	0.024	1.514(1.012–2.263)	0.045	0.284
C16185T	15(5.43)	3(1.23)	0.218(0.049–0.965)	0.046	0.198(0.044–0.896)	0.036	0.284
C16234T	7(2.33)	12(6.75)	3.038(1.102–8.386)	0.033	2.993(1.074–8.333)	0.037	0.284
C16260T	19(6.98)	4(1.84)	0.251(0.072–0.863)	0.029	0.246(0.070–0.860)	0.029	0.284
T16298C	42(15.89)	11(6.13)	0.347(0.168–0.712)	0.005	0.343(0.164–0.710)	0.005	0.181

**Figure 5:**
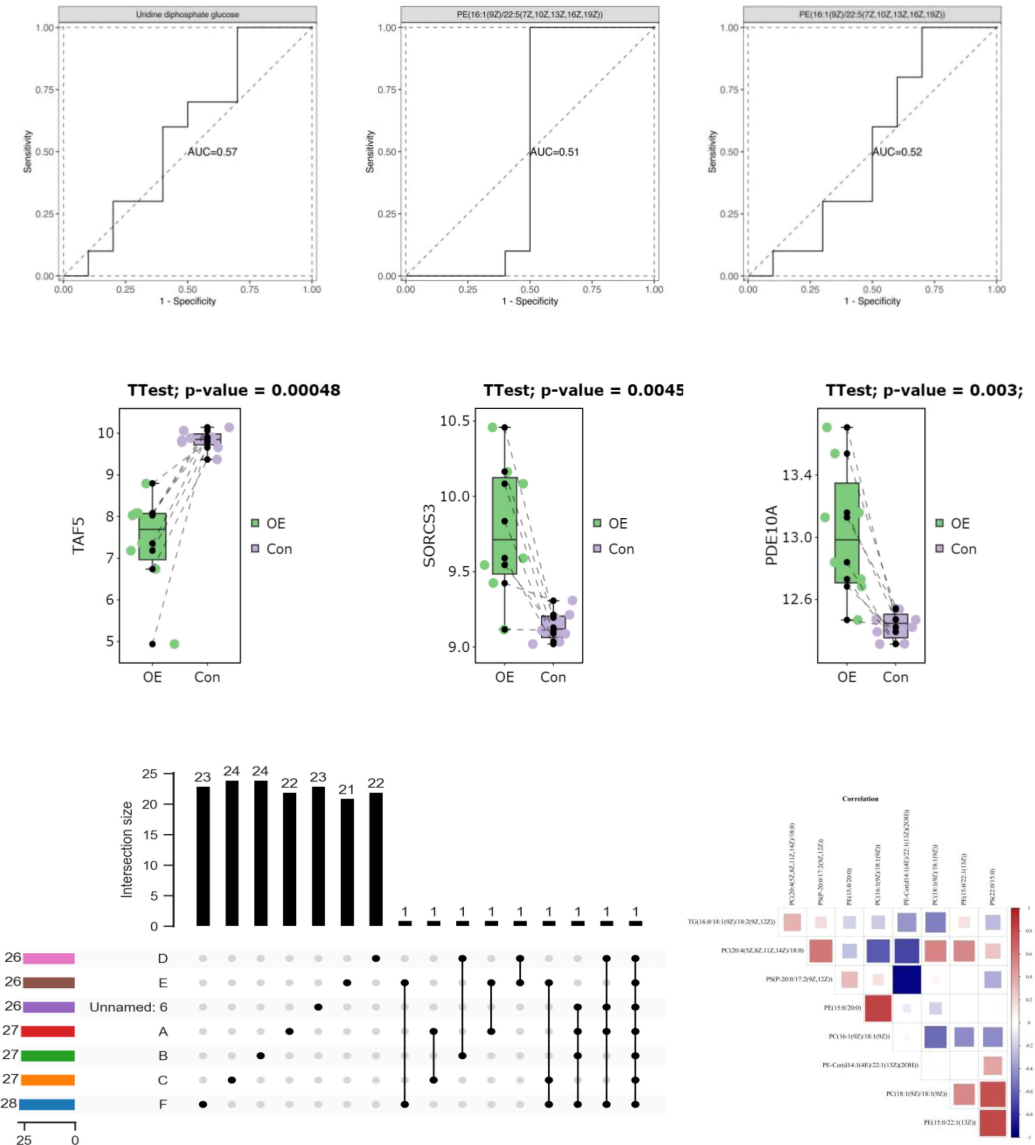
Association analysis of polymorphic loci in D-loop region with T levels.

The patients diagnosed with PCOS (PCOS) were categorized into two groups based on their testosterone levels: those with normal testosterone levels (T<2.64nmol/L) and those with hyperandrogenemia (T>2.64nmol/L). Subsequently, the polymorphic loci in the D-loop region were compared between these two groups. According to the data presented in **Table 5** and **Figure 5**, there was no longer a statistically significant difference in the expression of C16291T and T489C.

**Table 5:** Association analysis of polymorphic loci in D-loop region with T levels.

SNP_S_	T ≤ 2.64 nmol/L (n=358)	T >2.64nmol/L (n=63)	Before correction	*p*	After correction a	*p*	$P_{BH^{b}}$
	n(%)	n(%)	OR (95%CI)		OR (95%CI)>		
C489T	206(57.26)	28(42.86)	0.561(0.326–0.962)	0.037	0.566(0.327–0.975)	0.041	0.821
C16291T	8(1.96)	7(9.52)	5.279(1.712–16.271)	0.005	4.658(1.485–15.621)	0.009	0.329

**Figure 6:**
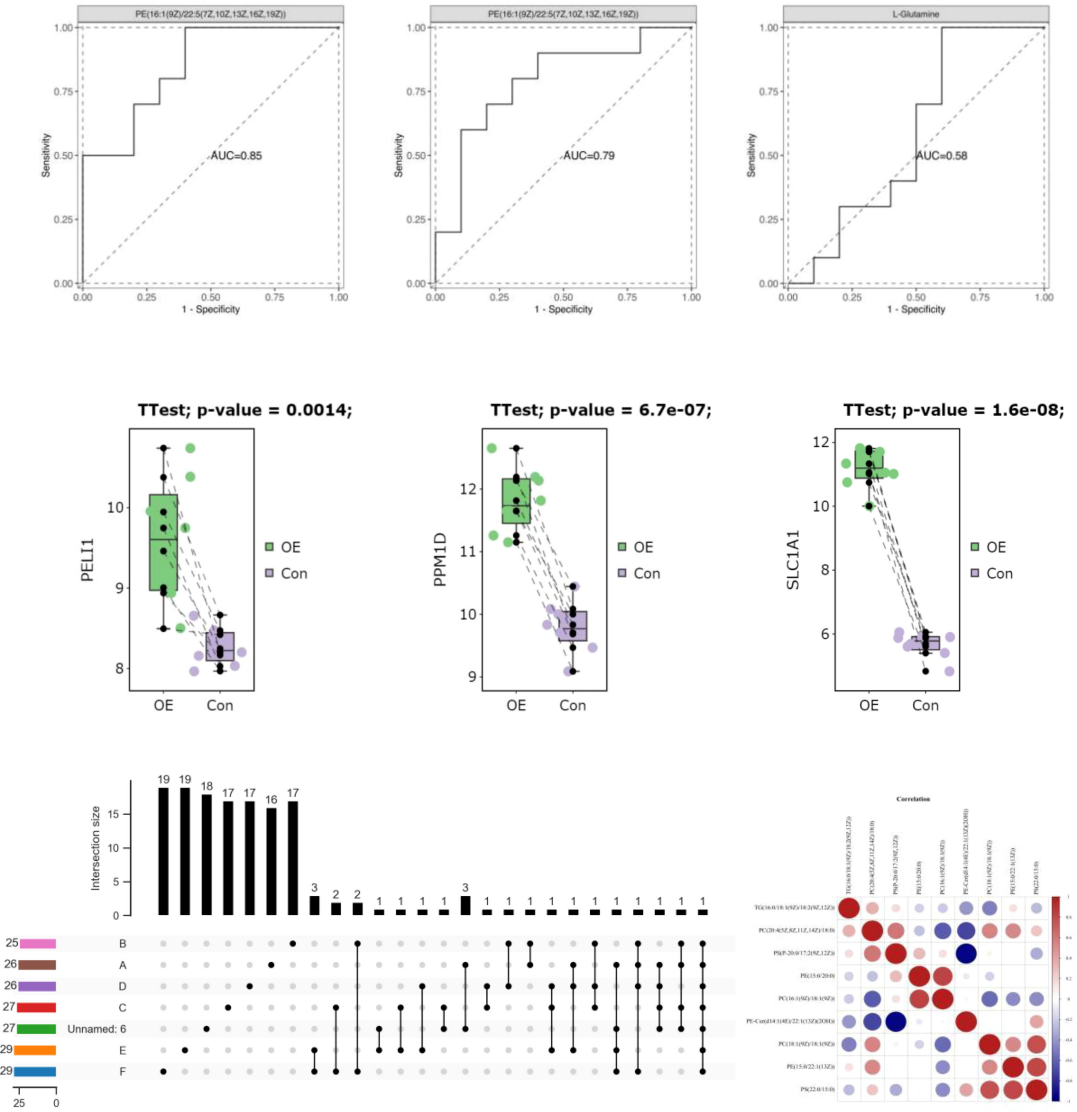
Association analysis of D-loop and region polymorphic loci with HOMA-IR.

As there is currently no standardized cutoff value for HOMA-IR in patients with PCOS (PCOS), the cut-off value of P7s-4.86, which represents the 75th quantile of HOMA-IR in PCOS patients, was employed as the designated threshold. However, the observed polymorphisms did not exhibit a significant correlation with HOMA-IR after adjusting for multiple testing using the FDR correction method, as indicated in **Table 6** and **Figure 6**.

**Table 6:** Association analysis of polymorphic loci in D-loop region with HOMA-IR.

SNP_S_	HOMA-IR ≤ 4.86 (n=316)	HOMA-IR ≤ 4.86 (n=105)	Before correction	*p*	After correction a	*p*	$p_{BH^{b}}$
	n(%)	n(%)	OR (95%CI)		OR (95%CI)		
C152T	99(31.02)	18(16.19)	0.431(0.243–0.761)	0.005	0.461(0.241–0.876)	0.019	0.431
523delAC	145(45.896)	36(33.33)	0.591(0.371–936)	0.026	649(0.384–1.092)	0.104	0.470
T16126C	6(1.58)	7(5.71)	3.771(1.126–12.619)	0.032	3.836(1.028–14.314)	0.046	0.431
A16203G	6(1.58)	7(5.71)	3.771(1.126–12.619)	0.032	1.765(0.462–6.743)	0.408	0.797
T16217C	28(8.54)	15(13.33)	1.648(0.828–3.274)	0.156	2.351(1.081–5.113)	0.032	0.431

**Figure 7:**
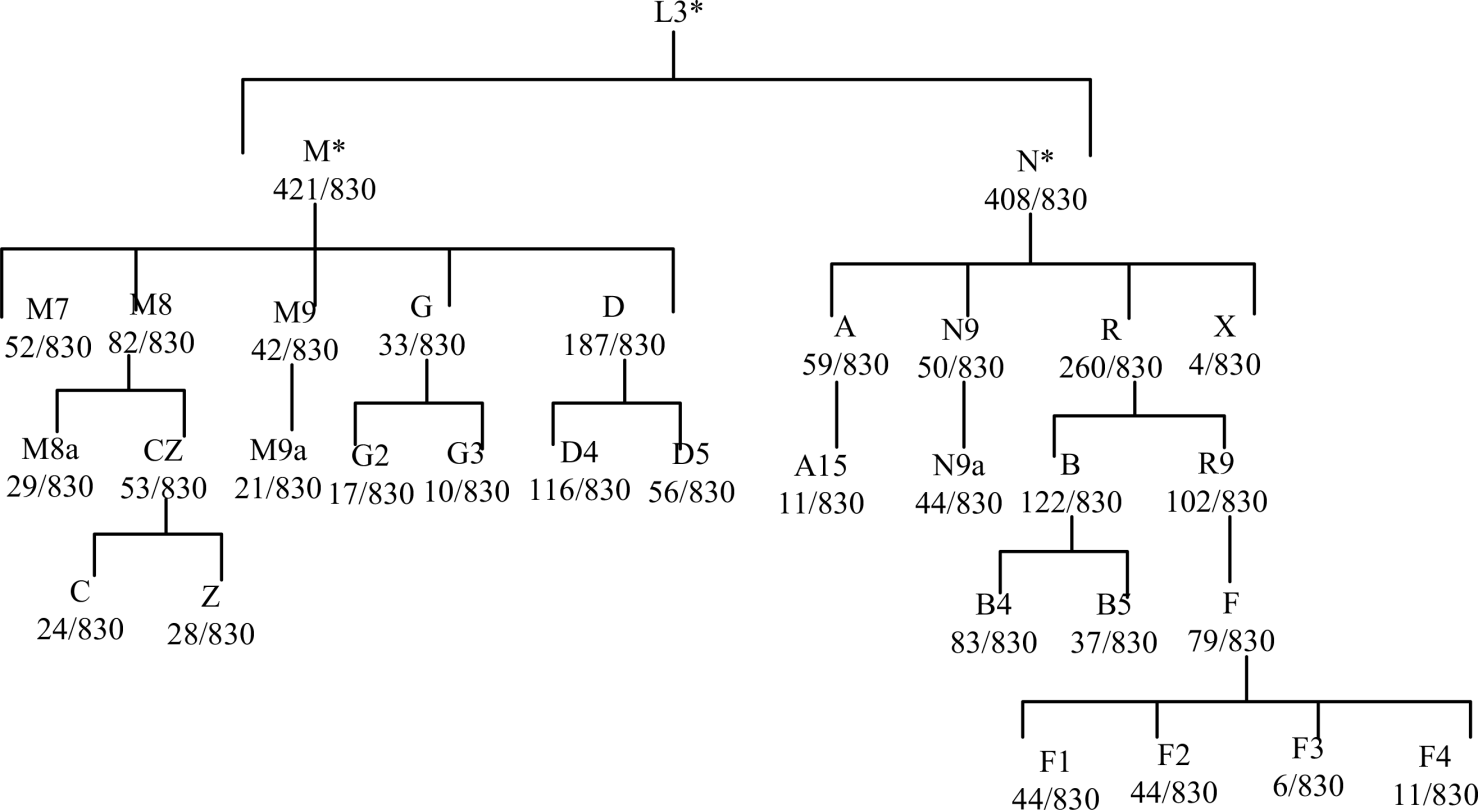
Phylogenetic dendrogram of mitochondrial haplotypes.

**Figure 8:**
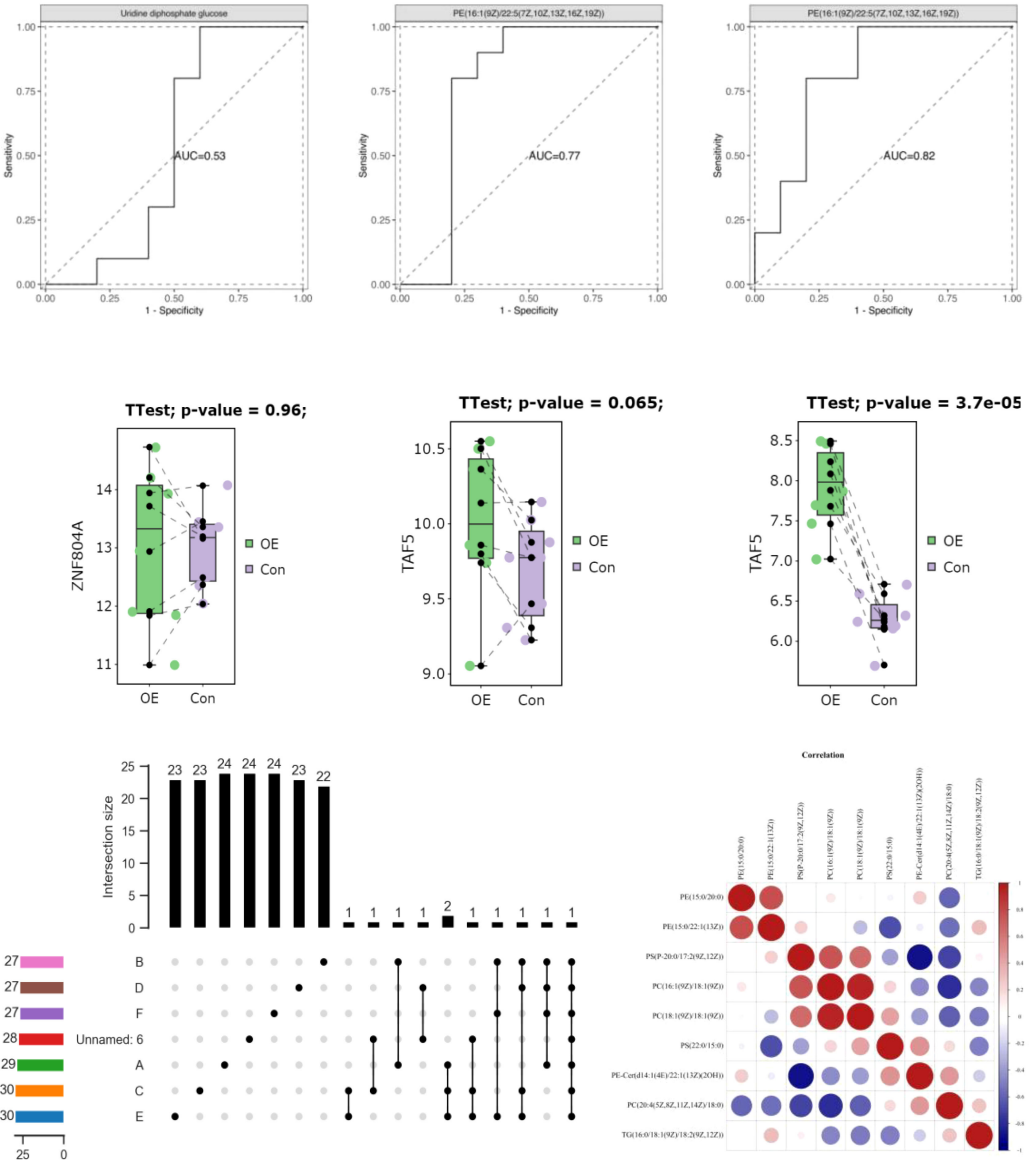
Association of A15 haplotype control.

Following adjustment for age and BMI, the results of the multivariate logistic regression analysis revealed a statistically significant association between haplotype A15 and the control group (*p*<0.01). In order to enhance the examination of the relationship between haplotypes and PCOS, the sub-haplotypes were consolidated into their respective major haplotype bundles using the Plant tree method available in the MITOMAP database. It is important to note that all haplotypes utilized in this analysis were sourced from the aforementioned database. All haplotypes originate from the L3 type, which signifies the common ancestry of human mitochondria. These haplotypes can be further classified into two major categories, namely, M and N. The M haplogroup can be further categorized into five sub-haplotypes, namely, M7, M8, M9, G, and D. The N type can be further classified into four distinct haplotypes, namely, A, N9, R, and X, which are arranged in a descending order. Among the subjects surveyed, it is observed that the R type exhibits the highest frequency of distribution, accounting for 260 out of 830 subjects. Following this, the D type is the second most prevalent, with a distribution of 187 out of 830 subjects. Lastly, the M8 type has the lowest frequency of distribution, representing 82 out of 830 subjects, as depicted in **Figure 7**. The M8 haplotype encompasses two primary sub-haplotypes, namely, M8a and CZ. Similarly, the G haplotype comprises two major sub-haplotypes, G2 and G3. The D haplotype consists of two prominent sub-haplotypes, D4 and D5. On the other hand, the R haplotype can be further subdivided into the B and R9 haplotypes, with B, D4, R9, and D5 being more prevalent within the population. The multivariate logistic regression analysis was conducted to examine the combined haplotypes. The results showed that haplotype A15 remained significantly associated with the control population (*p*=0.009). Haplotype X was found to be associated only with PCOS, while haplotype F3 was associated only with the control population. These findings are presented in **Figure 8** and **Table 7**.

**Table 7:** Correlation analysis of mitochondrial haplotypes and PCOS.

Haplotype	PCOS group (n=421)	Control group (n=409)	OR (95%CI)	$p^{a}$
L3	422 (100.00%)	410 (100.00%)		
M	205 (48.46%)	218 (53.06%)	0.890 (0.671–1.180)	0.417
M7	25 (5.70%)	29 (6.85%)	0.911 (0.511–1.625)	0.751
M8	46 (10.69%)	38 (19.05%)	1.195 (0.742–1.923)	0.467
M8a	16 (13.56%)	15 (3.42%)	1.068 (0.494–2.312)	0.870
CZ	31 (17.13%)	24 (15.62%)	1.257 (0.702–2.252)	0.445
C	14 (3.09%)	12 (12.69%)	1.143 (0.495–2.643)	0.757
Z	17 (3.80%)	13 (2.93%)	1.242 (0.558–2.763)	0.597
M9	18 (4.04%)	26 (6.11%)	0.656 (0.343–1.252)	0.201
M9a	8 (1.66%)	15 (3.42%)	0.494 (0.193–1.262)	0.141
G	13 (2.85%)	22 (5.13%)	0.561 (0.267–1.174)	0.125
G2	6 (1.19%)	13 (2.93%)	0.413 (0.139–1.227)	0.112
G3	7 (1.43%)	5 (0.98%)	1.433 (0.395–5.185)	0.586
D	91 (21.38%)	90 (21.76%)	1.047 (0.743–1.472)	0.798
D4	63 (14.73%)	55 (13.20%)	1.208 (0.804–1.812)	0.366
D5	26 (5.94%)	32 (7.58%)	0.852 (0.486–1.492)	0.575
N	218 (51.54%)	192 (46.70%)	1.136 (0.856–1.505)	0.380
A	26 (5.94%)	35 (8.31%)	0.671 (0.383–1.173)	0.162
A15	2 (0.24%)	11 (2.44%)	0.056 (0.006–0.486)	0.010
N9	27 (6.18%)	25 (5.87%)	0.935 (0.517–1.689)	0.823
N9a	23 (5.23%)	23 (5.38%)	0.870 (0.464–1.630)	0.664
R	142 (33.49%)	120 (29.10%)	1.165 (0.860–1.579)	0.326
B	67 (15.68%)	57 (13.69%)	1.156 (0.776–1.719)	0.480
B4	43 (9.98%)	42 (10.02%)	0.996 (0.623–1.589)	0.985
B5	24 (5.46%)	15 (3.42%)	1.611 (0.800–3.239)	0.183
R9	53 (12.35%)	51 (12.22%)	0.937 (0.609–1.438)	0.763
F	37 (8.56%)	44 (10.51%)	0.723 (0.445–1.173)	0.190
F1	24 (5.46%)	221 (5.13%)	1.025 (0.548–1.912)	0.942
F2	10 (2.14%)	9 (1.96%)	1.060 (0.378–2.966)	0.915
F3	1 (0%)	7 (1.47%)	/	1.000
F4	5 (0.95%)	8 (1.71%)	0.466 (0.129–1.667	0.241
X	5 (0.95%)	1 (0%)	/	1.000

**Figure 9:**
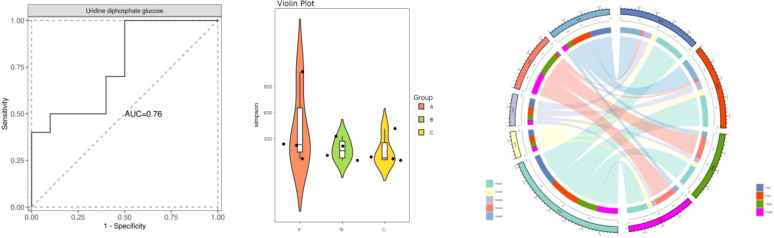
Comparative analysis of mtDNA copy number between PCOS and control.

The mtDNA (mtDNA) copy number exhibited an asymmetrical distribution in both the PCOS (PCOS) and control groups. Statistical analysis using the Mann–Whitney U test revealed that the median mtDNA copy number in the control group was 25.87 (interquartile range, 21.84–34.81), while the median mtDNA copy number in the PCOS group was 129.91 (interquartile range, 99.38–168.63). The mtDNA copy number was found to be significantly higher in individuals with PCOS (PCOS) compared to the control group (*p*<0.001). This information is presented in **Table 8** and **Figure 9**.

**Table 8:** Comparative analysis of mtDNA copy number between PCOS and control.

Project	PCOS group (n=168)	Control group (n=83)	*p*
Mt DNA copy	129.91(98.38,168.63)	25.87(21.84,34.81)	<0.002

## 4 Discussion

Typically, PCOS is diagnosed using a combination of clinical criteria, hormonal profiling, and imaging methods. Ultrasonography, specifically transvaginal ultrasound, is frequently used to evaluate ovarian morphology and detect the presence of ovarian lesions and follicles (39). Despite the fact that ultrasound imaging remains the most common method for diagnosing PCOS, there is a growing interest in investigating alternative methods (40). One such strategy could involve the use of ultrasound imaging via ViT-Patch, which have demonstrated promise in imaging malignant breast cancer (41). In addition, metabolic profiling of follicular fluid by nuclear magnetic resonance (NMR) has been used to gain insight into the physiological condition of PCOS patients (42). The enhanced sensitivity of hyperpolarized xenon NMR in conjunction with metal-organic frameworks holds promise for elucidating the fundamental mechanisms and metabolic changes associated with PCOS (43). Nevertheless, understanding the fundamental molecular basis of PCOS in patients is greatly facilitated by molecular techniques, such as evaluating mtDNA alterations. Collectively, these approaches advance our understanding of PCOS and its clinical phenotypes, particularly in the Chinese population. Thus, the purpose of this research was to examine the association between PCOS and mtDNA D-loop polymorphisms, haplotypes, and copy number in the Chinese population. Our results provide light on the possible links between these genetic variables and the prevalence of PCOS.

First, there were no statistically significant variations in age or prolactin (PRL) levels between the PCOS patients and the control group, according to the study’s demographic parameters. IR has been linked to PCOS, and individuals with PCOS have been shown to have considerably higher fasting insulin levels. This confirms what we already knew from looking at the pathophysiology of PCOS (**Table 1**; **Figure 1**). In a similar report, there were no statistically significant differences observed in the age and/or levels of follicle-stimulating hormone (FSH), PRL, estradiol (E2), progesterone (PRGE), or fasting glucose between patients belonging to women presenting with diagnosed hyperandrogenism, ovulatory dysfunction, and/or PCOS, as indicated by the clinical and biochemical characteristics of the participants (44).

We analyzed 505 nucleotide variations, including insertions, deletions, and substitutions, in the mtDNA D-loop region to investigate their potential role in PCOS. Due to the rarity of certain variations in the research population, we restricted our attention to 45 variants having a MAF of at least 5%. Interestingly, **Table 3** and **Figure 3** show that there is no association between these polymorphic loci and BMI in PCOS patients. These data implies that mtDNA D-loop mutations may not have a direct role in the association between PCOS and BMI. Our results imply that mtDNA D-loop polymorphisms may have a modest effect on BMI variance in the setting of PCOS, but it is important to keep in mind that BMI is a multifactorial characteristic impacted by both genetic and environmental factors.

Next, we looked at how the D-loop variations are connected to a major hormonal imbalance in PCOS: the LH/FSH ratio. Positive associations were found between a number of polymorphisms and elevated LH/FSH levels; these included C194T, 1A200G, 523delAC, and C16234T. After adjusting for age and BMI, however, the relationships no longer held (**Table 4**; **Figure 4**), suggesting that additional variables may affect LH/FSH levels in PCOS patients. Hormonal abnormalities in PCOS are the consequence of a dysregulation of the hypothalamic–pituitary–ovarian axis, which is itself the result of a combination of hereditary and environmental factors.

PCOS is also characterized by hyperandrogenemia, which is characterized by increased T levels. We looked at how D-loop variants and T levels are connected in women with PCOS. The expression of C16291T and T489C polymorphisms in PCOS patients with normal T levels and those with hyperandrogenemia did not vary significantly (**Table 5**; **Figure 5**). This indicates that these polymorphisms likely do not have a causal role in the androgen excess seen in PCOS. Hyperandrogenemia in PCOS is a complicated feature impacted by a wide range of genetic, hormonal, and environmental variables. The underlying genetic factors of hyperandrogenemia in PCOS need further investigation. A comparable study conducted on women from South India revealed that the A189G and D310 SNPs exhibited the strongest association with PCOS (28).

The HOMA-IR is a commonly used tool for measuring IR, which is a prevalent symptom of PCOS. The correlation between D-loop variations and HOMA-IR levels in PCOS patients was investigated. Once the FDR multiple testing correction was used, none of the previously identified variations linked with HOMA-IR (**Table 6**; **Figure 6**), suggesting that other genetic and environmental variables may play a more substantial role in insulin resistance in PCOS. However, we found that PCOS patients had considerably higher fasting insulin levels compared to the control group. This confirms earlier studies that have linked IR to the development of PCOS (44). Hyperinsulinemia, which is exacerbated by IR, promotes androgen synthesis in the ovaries, disrupts follicular growth, and impairs glucose metabolism. Increased insulin levels exacerbate the PCOS-related hormonal abnormalities by increasing LH synthesis and decreasing sex hormone-binding globulin (SHBG) production. In a separate study conducted on Iraqi women, it was found that those with PCOS exhibited notably elevated levels of LH, LH/FSH ratio, total testosterone (TT), fasting insulin, and HOMA-IR when compared to the control group (44).

In addition, we investigated whether or not mtDNA haplotypes play a part in the development of PCOS. Haplotypes, which are groups of related genetic variants that are passed down as a unit, may provide light on the ancestry and evolutionary history of certain populations. Major L3, M, N, and R haplotypes were found in the sample population. The haplotype A15 was shown to have a significant connection with the control group in a multivariate logistic regression analysis, suggesting a possible protective effect against PCOS (**Table 7**; **Figure 8**). In addition, the X haplotype was only discovered in PCOS individuals, indicating a possible link between the two. However, haplotype F3 was found only in the control group. These results suggest that certain haplotypes may influence PCOS risk and prevention. The complicated interaction between genetic variables and illness vulnerability is shown by our study’s connections between particular haplotypes and PCOS.

We also analyzed mtDNA copy number in PCOS patients and controls in addition to variations and haplotypes. Intriguingly, we found that the mtDNA copy number in PCOS patients was considerably greater than in controls. This discovery provides more evidence that mitochondrial function and biogenesis may be altered in PCOS. Energy metabolism is largely dependent on mitochondria, and mitochondrial malfunction may cause metabolic abnormalities, oxidative stress, and cellular dysfunction. The increased mtDNA copy number in PCOS patients (**Table 8**; **Figure 9**) may represent an adaptive response to mitochondrial malfunction or a compensatory reaction to the increased energy demands associated with PCOS. Conversely, the aforementioned study conducted in India utilized RT-PCR analysis to observe a notable reduction in mtDNA copy number among individuals diagnosed with PCOS in comparison to the control group. In addition, it was observed that individuals with PCOS who carried the D310 and 189G alleles exhibited a notably reduced mtDNA copy number in comparison to those who did not carry these alleles. The carriers of the D310 mutation also exhibited a notably increased ratio of LH/FSH (28). Correspondingly, in a separate study, it was observed that the mtDNA copy numbers were significantly lower in the group of individuals with PCOS, regardless of their diabetic status (44).

Overall, our results provide new light on how mtDNA D-loop mutations, haplotypes, and copy number contribute to PCOS prevalence in the Chinese population. We detected possible protective or susceptibility effects of various haplotypes and observed significant relationships between certain variations and hormonal imbalances (e.g., LH/FSH ratio). In addition, mitochondrial dysfunction may play a role in PCOS pathogenesis because of the increased mtDNA copy number in these individuals. These results improve our knowledge of the genetic basis of PCOS and may have future applications in clinical management and individualized treatment strategies. One essential strategy for the management of this condition involves the utilization of probes capable of identifying the modified mitochondria within abnormal cells (45, 46). Subsequently, these probes facilitate the targeted delivery of precise pharmaceutical agents and biological substances to the affected cells through the employment of nanocarriers (47).

To confirm and broaden these results in broader and more varied groups, further study is needed.

## 5 Conclusion

In conclusion, our research elucidates the significance of mtDNA D-loop polymorphisms, haplotypes, and copy quantity in the occurrence of PCOS in China. We found possible links between certain genetic variants and hormonal abnormalities, suggesting their participation in the pathogenesis of PCOS. In addition, the increased mtDNA copy number in PCOS suggests altered mitochondrial function and biogenesis, as shown by our results. These findings improve our comprehension of PCOS’s genetic underpinnings and its clinical consequences. To confirm and generalize these results across varied groups, more research is necessary. Overall, this study deepens our understanding of PCOS and lays the groundwork for designing individualized treatments for those who suffer from it.

## Data availability statement

The original contributions presented in the study are included in the article/supplementary material. Further inquiries can be directed to the corresponding authors.

## Author contributions

The study’s design, data collection, data analysis, and manuscript composition were developed by YC, W-jW, L-wX, X-jZ, JW, X-yX, RuZ, and RoZ. The final version of the manuscript was collaboratively developed and subsequently endorsed by all contributing authors. All authors contributed to the article and approved the submitted version.

## References

[1] Lauritsen MP Bentzen J Pinborg A Loft A Forman J Thuesen L. The prevalence of polycystic ovary syndrome in a normal population according to the Rotterdam criteria versus revised criteria including anti-Müllerian hormone. Hum Reprod (2014) 29(4):791–801. doi: 10.1093/humrep/det469 24435776

[2] Fakhoury H Tamim H Ferwana M Siddiqui IA Adham M Tamimi W. Age and BMI adjusted comparison of reproductive hormones in PCOS. J Family Med primary Care (2012) 1(2):132. doi: 10.4103/2249-4863.104984 24479022 PMC3893977

[3] Holte J Bergh T Berne C Lithell H. Serum lipoprotein lipid profile in women with the polycystic ovary syndrome: relation to anthropometric, endocrine and metabolic variables. Clin Endocrinol (1994) 41(4):463–71. doi: 10.1111/j.1365-2265.1994.tb02577.x 7955457

[4] Wild RA Rizzo M Clifton S Carmina E. Lipid levels in polycystic ovary syndrome: systematic review and meta-analysis. Fertility sterility (2011) 95(3):1073–9. e11. doi: 10.1016/j.fertnstert.2010.12.027 21247558

[5] Zhou S Hua R Quan S. N6-methyladenosine regulator-mediated methylation modification patterns and immune infiltration characterization in Polycystic Ovary Syndrome (PCOS). J Ovarian Res (2023) 16(1):73. doi: 10.1186/s13048-023-01147-9 37046273 PMC10091541

[6] Zhang S Deng W Liu Q Wang P Yang W Ni W. Altered m(6) A modification is involved in up-regulated expression of FOXO3 in luteinized granulosa cells of non-obese polycystic ovary syndrome patients. J Cell Mol Med (2020) 24(20):11874–82. doi: 10.1111/jcmm.15807 PMC7578862 32869942

[7] Li L Zhu J Ye F Duan Z Zhou J Huang Z. Upregulation of the lncRNA SRLR in polycystic ovary syndrome regulates cell apoptosis and IL-6 expression. Cell Biochem Funct (2020) 38(7):880–5. doi: 10.1002/cbf.3507 PMC7586972 31999854

[8] Liu S Li Q Chen K Zhang Q Li G Zhuo L. The emerging molecular mechanism of m6A modulators in tumorigenesis and cancer progression. Biomedicine Pharmacotherapy (2020) 127:110098. doi: 10.1016/j.biopha.2020.110098 32299028

[9] Li C Lin L Zhang L Xu R Chen X Ji J. Long noncoding RNA p21 enhances autophagy to alleviate endothelial progenitor cells damage and promote endothelial repair in hypertension through SESN2/AMPK/TSC2 pathway. Pharmacol Res (2021) 173:105920. doi: 10.1016/j.phrs.2021.105920 34601081

[10] Rahimpour A Heidarzadehpilehrood R Abdollahi S Ranjbari H Shams Z Ghasemi SA. A comprehensive bioinformatic analysis revealed novel MicroRNA biomarkers of Parkinson's disease. Cell Biol Int (2022) 46(11):1841–51. doi: 10.1002/cbin.11869 36098337

[11] Chu C Zhang QC Da Rocha ST Flynn RA Bharadwaj M Calabrese JM. Systematic discovery of Xist RNA binding proteins. Cell (2015) 161(2):404–16. doi: 10.1016/j.cell.2015.03.025 PMC4425988 25843628

[12] Murri M Luque-Ramírez M Insenser M Ojeda-Ojeda M Escobar-Morreale HF. Circulating markers of oxidative stress and polycystic ovary syndrome (PCOS): a systematic review and meta-analysis. Hum Reprod Update (2013) 19(3):268–88. doi: 10.1093/humupd/dms059 23303572

[13] Mohammadi M. Oxidative stress and polycystic ovary syndrome: a brief review. Int J Prev Med (2019) 10(1):86–92. doi: 10.4103/ijpvm.IJPVM_576_17 PMC6547785 31198521

[14] Rigotto G Basso E. Mitochondrial dysfunctions: a thread sewing together Alzheimer’s disease, diabetes, and obesity. Oxid Med Cell Longevity (2019) 2019:1–16. doi: 10.1155/2019/7210892 PMC6604285 31316720

[15] Stoneking M. Hypervariable sites in the mtDNA control region are mutational hotspots. Am J Hum Genet (2000) 67(4):1029–32. doi: 10.1086/303092 PMC1287875 10968778

[16] Taanman J-W. The mitochondrial genome: structure, transcription, translation and replication. Biochim Biophys Acta (BBA)-Bioenergetics (1999) 1410(2):103–23. doi: 10.1016/S0005-2728(98)00161-3 10076021

[17] Govatati S Deenadayal M Shivaji S Bhanoori M. Mitochondrial displacement loop alterations are associated with endometriosis. Fertility sterility (2013) 99(7):1980–6. e9. doi: 10.1016/j.fertnstert.2013.02.021 23490167

[18] Tipirisetti NR Govatati S Pullari P Malempati S Thupurani MK Perugu S. Mitochondrial control region alterations and breast cancer risk: a study in South Indian population. PloS One (2014) 9(1):e85363. doi: 10.1371/journal.pone.0085363 24497926 PMC3907410

[19] Zhuo G Ding Y Feng G Yu L Jiang Y. Analysis of mitochondrial DNA sequence variants in patients with polycystic ovary syndrome. Arch gynecology obstetrics (2012) 286:653–9. doi: 10.1007/s00404-012-2358-7 22546954

[20] Zhuo G Feng G Leng J Yu L Jiang Y. A 9-bp deletion homoplasmy in women with polycystic ovary syndrome revealed by mitochondrial genome-mutation screen. Biochem Genet (2010) 48:157–63. doi: 10.1007/s10528-009-9308-5 20094848

[21] Li X Ji D Marley JL Zou W Deng X Cao Y. Association between mitochondrial DNA D-loop region polymorphisms and endometriosis in a Chinese population. J assisted Reprod Genet (2020) 37(9):2171–9. doi: 10.1007/s10815-020-01853-z PMC7492337 32535813

[22] Jansen RP Burton GJ. Mitochondrial dysfunction in reproduction. Mitochondrion (2004) 4(5-6):577–600. doi: 10.1016/j.mito.2004.07.038 16120416

[23] Fernández-Silva P Enriquez JA Montoya J. Replication and transcription of mammalian mitochondrial DNA. Exp Physiol (2003) 88(1):41–56. doi: 10.1113/eph8802514 12525854

[24] Montier LLC Deng JJ Bai Y. Number matters: control of mammalian mitochondrial DNA copy number. J Genet Genomics (2009) 36(3):125–31. doi: 10.1016/S1673-8527(08)60099-5 PMC4706993 19302968

[25] Lee H-C Lu C-Y Fahn H-J Wei Y-H. Aging-and smoking-associated alteration in the relative content of mitochondrial DNA in human lung. FEBS Lett (1998) 441(2):292–6. doi: 10.1016/S0014-5793(98)01564-6 9883902

[26] Verma M Naviaux RK Tanaka M Kumar D Franceschi C Singh KK. Meeting report: mitochondrial DNA and cancer epidemiology. AACR (2007) 67(2):437–439. doi: 10.1158/0008-5472.CAN-06-4119 17213255

[27] Lee S-H Chung D-J Lee H-S Kim T-J Kim M-H Jeong HJ. Mitochondrial DNA copy number in peripheral blood in polycystic ovary syndrome. Metabolism (2011) 60(12):1677–82. doi: 10.1016/j.metabol.2011.04.010 21676419

[28] Reddy TV Govatati S Deenadayal M Sisinthy S Bhanoori M. Impact of mitochondrial DNA copy number and displacement loop alterations on polycystic ovary syndrome risk in south Indian women. Mitochondrion (2019) 44:35–40. doi: 10.1016/j.mito.2017.12.010 29278759

[29] Van Oven M Kayser M. Updated comprehensive phylogenetic tree of global human mitochondrial DNA variation. Hum Mutat (2009) 30(2):E386–E94. doi: 10.1002/humu.20921 18853457

[30] Nacmias B Reitz C Arendt T. Early clinical and molecular detection of Alzheimer's disease. Hindawi (2011) 2011:1. doi: 10.4061/2011/818639 PMC3216385 22114746

[31] Chinnery PF Gomez-Duran A. Oldies but goldies mtDNA population variants and neurodegenerative diseases. Front Neurosci (2018) 682. doi: 10.3389/fnins.2018.00682 PMC6194173 30369864

[32] Wang L Hu J Zhou H. Macrophage and adipocyte mitochondrial dysfunction in obesity-induced metabolic diseases. World J Men's Health (2021) 39(4):606. doi: 10.5534/wjmh.200163 33151047 PMC8443980

[33] Jersin RÅ Tallapragada DSP Madsen A Skartveit L Fjære E McCann A. Role of the neutral amino acid transporter SLC7A10 in adipocyte lipid storage, obesity, and insulin resistance. Diabetes (2021) 70(3):680–95. doi: 10.2337/db20-0096 33408126

[34] Lim S Deaver JW Rosa-Caldwell ME Lee DE Morena da Silva F Cabrera AR. Muscle miR-16 deletion results in impaired insulin sensitivity and contractile function in a sex-dependent manner. Am J Physiology-Endocrinology Metab (2022) 322(3):E278–E92. doi: 10.1152/ajpendo.00333.2021 PMC8897019 35068192

[35] Wang Y Yang Q Wang H Zhu J Cong L Li H. NAD+ deficiency and mitochondrial dysfunction in granulosa cells of women with polycystic ovary syndrome. Biol Reprod (2021) 105(2):371–80. doi: 10.1093/biolre/ioab078 34056649

[36] Lipina C Macrae K Suhm T Weigert C Blachnio-Zabielska A Baranowski M. Mitochondrial substrate availability and its role in lipid-induced insulin resistance and proinflammatory signaling in skeletal muscle. Diabetes (2013) 62(10):3426–36. doi: 10.2337/db13-0264 PMC3781443 23733201

[37] Pinelis I Pinelis V. Brain insulin resistance: focus on insulin receptor-mitochondria interactions. (2021) 11(3):262–78. doi: 10.20944/preprints202103.0401.v1 PMC8005009 33810179

[38] Keni R Sekhar A Gourishetti K Nayak PG Kinra M Kumar N. Role of statins in new-onset diabetes mellitus: the underlying cause, mechanisms involved, and strategies to combat. Curr Drug Targets (2021) 22(10):1121–8. doi: 10.2174/1389450122666210120125945 33494673

[39] Denny A Raj A Ashok A Ram CM George R eds. i-hope: Detection and prediction system for polycystic ovary syndrome (pcos) using machine learning techniques. In: TENCON 2019-2019 IEEE Region 10 Conference (TENCON). IEEE. Held at the Grand Hyatt Kochi Bolgatti, Kerala, India.

[40] Allemand MC Tummon IS Phy JL Foong SC Dumesic DA Session DR. Diagnosis of polycystic ovaries by three-dimensional transvaginal ultrasound. Fertility sterility (2006) 85(1):214–9. doi: 10.1016/j.fertnstert.2005.07.1279 16412756

[41] Feng H Yang B Wang J Liu M Yin L Zheng W. Identifying malignant breast ultrasound images using ViT-patch. Appl Sci (2023) 13(6):3489. doi: 10.3390/app13063489

[42] Castiglione Morelli MA Iuliano A Schettini SCA Petruzzi D Ferri A Colucci P. NMR metabolic profiling of follicular fluid for investigating the different causes of female infertility: a pilot study. Metabolomics Off J Metabolomic Soc (2019) 15(2):19. doi: 10.1007/s11306-019-1481-x 30830455

[43] Zeng Q Bie B Guo Q Yuan Y Han Q Han X. Hyperpolarized Xe NMR signal advancement by metal-organic framework entrapment in aqueous solution. Proc Natl Acad Sci (2020) 117(30):17558–63. doi: 10.1073/pnas.2004121117 PMC7395552 32661173

[44] Saeed N Hamzah IH Al-Gharrawi SAR. Polycystic ovary syndrome dependency on mtDNA mutation; copy Number and its association with insulin resistance. BMC Res Notes (2019) 12(1):455. doi: 10.1186/s13104-019-4453-3 31340838 PMC6657173

[45] Jin T Cui M Wu D Zhu W Xu Y Qian X. NCL-based mitochondrial-targeting fluorescent probe for the detection of Glutathione in living cells. Chin Chem Lett (2021) 32(12):3899–902. doi: 10.1016/j.cclet.2021.06.033

[46] Bu D Wang Y Wu N Feng W Wei D Li Z. A mitochondrial-targeted ratiometric probe for detecting intracellular H2S with high photostability. Chin Chem Lett (2021) 32(5):1799–802. doi: 10.1016/j.cclet.2020.12.044

[47] Huang L Sun Z Shen Q Huang Z Wang S Yang N. Rational design of nanocarriers for mitochondria-targeted drug delivery. Chin Chem Lett (2022) 33(9):4146–56. doi: 10.1016/j.cclet.2022.02.047

